# Development of a fuzzy model for differentiating peanut plant from broadleaf weeds using image features

**DOI:** 10.1186/s13007-020-00695-1

**Published:** 2020-11-16

**Authors:** Adel Bakhshipour, Hemad Zareiforoush

**Affiliations:** Department of Agricultural Mechanization Engineering, Faculty of Agricultural Sciences, University of Guilan, Rasht, Iran

**Keywords:** Fuzzy logic, Image processing, Peanut, Wavelet transform, Weed detection

## Abstract

A combination of decision tree (DT) and fuzzy logic techniques was used to develop a fuzzy model for differentiating peanut plant from weeds. Color features and wavelet-based texture features were extracted from images of peanut plant and its three common weeds. Two feature selection techniques namely Principal Component Analysis (PCA) and Correlation-based Feature Selection (CFS) were applied on input dataset and three Decision Trees (DTs) including J48, Random Tree (RT), and Reduced Error Pruning (REP) were used to distinguish between different plants. In all cases, the best overall classification accuracies were achieved when CFS-selected features were used as input data. The obtained accuracies of J48-CFS, REP-CFS, and RT-CFS trees for classification of the four plant categories namely peanut plant, Velvetleaf, False daisy, and Nicandra, were 80.83%, 80.00% and 79.17% respectively. Along with these almost low accuracies, the structures of the decision trees were complex making them unsuitable for developing a fuzzy inference system. The classifiers were also used for differentiating peanut plant from the group of weeds. The overall accuracies on training and testing datasets were respectively 95.56% and 93.75% for J48-CFS; 92.78% and 91.67% for REP-CFS; and 93.33% and 92.59% for RT-CFS DTs. The results showed that the J48-CFS and REP-CFS were the most appropriate models to set the membership functions and rules of the fuzzy classifier system. Based on the results, it can be concluded that the developed DT-based fuzzy logic model can be used effectively to discriminate weeds from peanut plant in the form of machine vision-based cultivating systems.

## Background

Despite the remarkable progress made in agricultural industry in recent years, weed management is still a challenging and complex problem.

There are mainly three methods for weed control in agricultural fields, namely, manual removal, mechanical hoeing, and chemical weed control. Traditional manual removal of weeds is still a common practice in the peanut fields. Hand hoeing is a very effective operation which is carried out properly. However, it is a tedious, extremely labor-intensive and time consuming operation with adverse health effects. Furthermore, manual weeding through hand tools can only be employed in small-scale farming or in home gardens and it is not a good practice in large scale cultivation [1, 2].

Even though mechanical weeding using inter-row cultivators is frequently used to remove the weeds between rows [3], the presence of weeds within crop rows and very closed to the main plant, makes it very difficult to eliminate intra-row weeds by mechanical cultivation implements where mechanical weed control in close proximity to the crop plant may damage the crop [4].

The most widespread method in weeds elimination is using herbicides. Conventionally, herbicides are uniformly applied to all parts of a farm even if there are no weeds in some certain parts of the farm. Increasing the cost of agriculture, environmental pollution, and negative effect on the human health are the major drawbacks associated with uniform application of herbicides [5, 6]. Variable rate application of herbicides, based on the presence and intensity of weeds in different parts of fields, can make it possible to optimally use chemicals, which leads to reduced cost and minimal environmental contamination.

Either mechanical destroying of intra-row weeds without damaging to crop plants, or variable rate spraying, both primarily necessitate it to precisely detect and locate weeds in crop rows. Site-specific weed management is an essential part of the progress towards economically and environmentally sustainable weed management [7]. Precision weed control within a field majorly requires information about weed distribution [8].

The first step towards determining weed distribution and density is to detect and segregate weeds from main crop. Computer vision and image processing techniques provide the capability to detect the desired objects in digital images or videos that are acquired from a scene. The acceptance and utilization of computer vision systems has been rapid and widespread. Along with other fields, the use of image processing techniques and image-extracted data for agro/food-informatics applications, have been widely investigated by researchers and reported in the literature [9–12]. Also, Studies on crop and weed detection using visible range image processing approaches has also been interested and conducted by many researchers [3, 13–16].

While there are literature reporting successful application of multi/hyper spectral image analysis for crop and weed detection [17–19], high quality non-visible spectrum imaging systems are generally more expensive and not-affordable to access—rather than visible ones—for researchers and farmers [20, 21]. Therefore, Optimizing the RGB image processing techniques for weed/crop detecting, is still a challenge. Some of the visible range image processing techniques are color, texture, and wavelet-based multi-resolution analysis.

Discrete Wavelet Transform (DWT) is a multiresolution image analysis tool which decomposes images into low and high frequency subbands by applying successive high-pass and low-pass filtering [22]. By implementing DWT on an image, approximation and details coefficients are obtained. Approximation coefficient subimages contain low frequencies which express the whole global trend of the image, whereas the detail coefficient subimages contain higher frequencies that express the local steep changes. Therefore, DWT is one of the powerful texture feature analysis techniques [23, 24]. Comprehensive information about wavelet transform and its applications in images, are provided by Vyas et al. [25] and Kolekar et al. [26]. Applications of DWT were reported for several agro-food related areas [11, 24, 27, 28].

By using a suitable classifier, different objects in images can be distinguished into separate groups based on extracted features. Decision trees and Fuzzy logic models are two of the popular learning techniques in computer vision systems.

DTs are hierarchical classifiers that predict class membership by heuristically selecting the most relevant attributes and searching for if-then-else rules that best split the samples into correct classes [29, 30]. DTs have shown to obtain classification performance close to or even outperforming other state-of-the-art methods [31].

On the other side, fuzzy logic theory, which was originally introduced by [32], is a problem solving tool that deals with approximate reasoning rather than fixed and exact reasoning [33]. Thus, distinct from conventional techniques, fuzzy sets are capable of dealing with vague, ambiguous and imprecise data [34]. The basic idea of fuzzy logic is to replace the “crisp” truth values 1 and 0 by a degree of truth in the interval of 0 to 1 [35]. Fuzzy logic can help in the site-specific application of herbicides based on outputs from an image processing system, either in the real time or by the image-based weed maps [36]. Herrera et al. [37] applied a fuzzy decision-making method for discrimination between grasses and broad-leaved weeds based on shape descriptors. The best classification accuracy was reported to be 92.9%. An equal accuracy of fuzzy classifier was reported by Sujaritha et al. [38] for classification of sugarcane crop among nine different weed species based on leaf textures.

It should be also noted that, in most cases of weed/crop classification processes, due to close similarity of weeds and main crop, very different image-based information must be investigated to find those set of features that satisfactorily differentiate between plant types. Furthermore, due to exponentially increasing the number of fuzzy logic rules by increasing the input variables, it is difficult to adjust fuzzy rules when there is a large amount of input variables [39]. Moreover, depending on the result of human adjustment, there is no guarantee to yield the optimal solution [40]. Therefore, if the use of fuzzy system for such a complex problem is intended, there is a need for invoking strategies to effectively incorporate numerous types of image-extracted features into the fuzzified weed detection system. By combining the DT and fuzzy logic approaches, the automatically selected features and generated rules by DT can be applied for constructing and tuning fuzzy classifier.

Combination of fuzzy learning algorithms with DT-based approaches takes advantages of the smooth decision that is obtained by a fuzzy classifier. Application of DT based fuzzy models benefits from advantages of fuzzy systems while still maintaining the benefits of DT classifiers such as comprehensibility and interpretability. It was stated by Ayed et al. [41] that the non-fuzzy decision trees are noise sensitive, and the process of decision depends on the border values, meanwhile, the fuzzy decision trees are more robust to the noise, and eliminate the border problems because of having linguistic outputs. DT-based fuzzy systems have been used for classification problems in agriculture-related studies [34, 42, 43].

Reviewing the literature, although good accuracies have been reported for weed/crop classification when applying several techniques [15, 44–46], however these researches don’t provide information about rules, membership functions, and antecedent, to be useful for developing a fuzzy weed detection system. Therefore, the aim of this study was to investigate the capability of DT-based fuzzy logic system for classification of some broadleaf plants in peanut fields using image extracted color and wavelet information.

## Material and methods

### Image acquisition

**Fig. 1:**
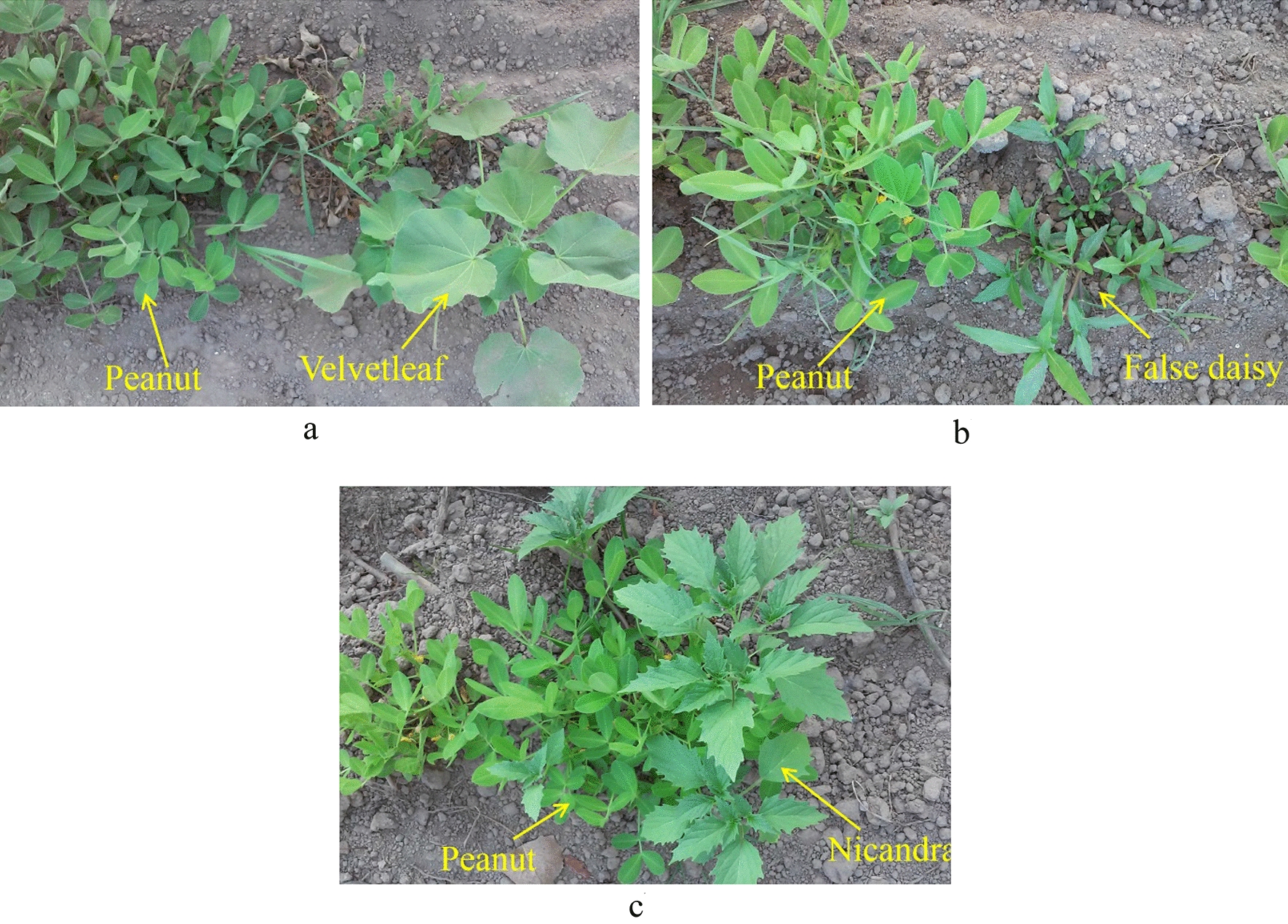
Sample images of studied plants; **a** Peanut and Velvetleaf, **b** Peanut and False daisy, **c** Peanut and Nicandra

Images of peanut plant (*Arachis hypogaea*) and three common broadleaf weeds in peanut fields namely Velvetleaf (*Abutilon theophrusti*), False daisy (*Eclipta alba*), and Nicandra (*Nicandra physalodes*) were acquired using an affordable cell phone camera having a resolution of 2240 × 1344 pixels. The images were captured vertically downwards from a distance of 40 cm above the crop rows. A white cotton sunshade was used to let the image acquisition scene be illuminated by diffused sunlight and to prevent leaf shadows on each other. A total number of 100 images were prepared for this study. Figure 1 shows sample images of the studied plants.

### Feature extraction

I order to extract image wavelet information of four mentioned plant types, fifty blocks of 100 × 100 pixels were manually cropped from images of each of plant (totally; 50 × 4 = 200 image blocks) using Photoshop CS6 software (Adobe Systems, USA). Original field-captured Images were loaded in Photoshop and the blocks were extracted carefully from different regions of the desired plants using “*marquee tool*” in the Photoshop toolbar, without any change in resolution and color values of images. Region of interest selection using different region selection tools of Photoshop software have been reported in several image processing related researches [44, 47, 48]. The cropped blocks were introduced into image processing toolbox of MATLAB software version 2018a (The MathWorks, USA).

The prepared image blocks were transferred from RGB into HSV and L*a*b color spaces using conversion functions of “*rgb2hsv*” and “*rgb2lab*” in MATLAB software [27, 49, 50], and the average and standard deviation values of Red, Green, Blue, Hue, Saturation, Value, Luminance, a* and b* color components were extracted.

In this study, Gray-Level Co-occurrence Matrix (GLCM) algorithm, which is a statistical texture analysis approach, was employed for extracting texture features from images. In order to extract such features, the color blocks were converted to gray-scale images using “*rgb2gray*” function and the Gray Level Co-occurrence Matrices (GLCMs) were contracted from gray blocks. GLCM represents the distribution of co-occurring values at given offset over an image. In this study, GLCMs were calculated for a spatial distance of one pixel in four different directions (0°, 45°, 90°, and 135°). Average of four resulting GLCM was calculated and used for feature extraction from the related-grayscale image. In this study 16 different GLCM-based texture features namely; entropy, energy, inertia, correlation, homogeneity, dissimilarity, sum of squares, sum of averages, sum of variances, sum of entropies, difference variance, difference entropy, cluster shade, cluster prominence, inverse difference moment, and maximum probability were calculated from GLCMs and used for plant classification. These features have been previously described in detail and used by several researchers [44, 51–55].

In the other part of the study, one-level two-directional Haar wavelet transform was applied on the gray-scale images of blocks and four sub-images were derived. The resulted subimages were approximation, horizontal details, vertical details, and diagonal details which are also called LL (Low–Low), LH (Low–High), HL (High–Low), and HH (High–High) sub-bands respectively. Haar wavelet, which is a same wavelet as Daubechies db1, known as the first, the simplest, and the fastest wavelet type [56].

After obtaining the wavelet subbands, the above-mentioned texture features were also extracted from each of the subband images. Therefore 64 wavelet-texture features were extracted for each block (16 texture features × 4 wavelet subimages) and used for classification of peanut and weeds.

### Feature selection

There was a very large number of input features to be fed into the classifiers (18 color features + 16 texture features + 64 wavelet based texture features = 98 input features). Large dimension of input data can affect the performance of classifiers as the most of pattern recognition techniques are originally not designed to cope with large amounts of irrelevant features [57]. Therefore, two feature selection methods were applied to input feature dataset in order to identify the most relevant features (or feature vectors) and to eliminate the redundant to obtain a higher classification performance: Principal Component Analysis (PCA), which is an unsupervised feature selection method, and Correlation-based Feature Selection (CFS), which is a supervised feature selection method [58, 59].

### Decision tree (DT)

Three types of DTs, including J48, Reduced Error Pruning (REP), and Random Tree (RT), were applied for distinguishing different plants. The J48 DT-inducing algorithm is an implementation of the well-known C4.5 DT in WEKA software [60]. The C4.5 tree which is known as one of top 10 data mining algorithms, chooses appropriate features and nodes based on the information gain ratios [61, 62]. REP tree classifier is a fast DT learner that builds a tree based on information gain with entropy and prunes it using reduced-error pruning [63]. RT is a DT that it’s nodes are constructed using randomly chosen attributes and the class probabilities on each node are based on back fitting with no pruning [64].

### DT Performance evaluation

The original datasets were splitted randomly into three subsets including training (60%), cross-validation (20%), and testing (20%) data. The most successful DT models were selected preliminary based on having the highest value of classification accuracy on training dataset. Classification accuracy shows that how close to the observed data are the predictions given by a classifier. This criterion was calculated using Eq. 1 [65].

1 $$Accuracy=\frac{TP+TN}{TP+TN+FP+FN}$$

where *TP*, *TN*, *FP*, and *FN* denote, respectively, true positive, true negative, false positive, and false negative measures in the confusion matrix of the classifier. In this equation, true positive was the number of positive samples (e.g. peanut plant) that classified as positive. True negative was the number of negative samples (e.g. weeds) that classified as negative. False positive was the number of negative samples classified as positive, and false negative was number of positive samples classified as negative. The accuracy metric indicates the overall effectiveness of the system considering positive and negative samples [66].

In addition to classification accuracy, two other statistics were calculated for DT models to be used in the probable cases in which there were DT models having similar classification accuracies. These two statistical indicators were Cohen's kappa coefficient and Root Mean Squared Error (RMSE), that were measured on training dataset and used to evaluate the performance of the developed classifiers. Kappa is a statistic which measures inter-rater agreement for categorical items. It is generally thought to be a more robust measure than simple percent agreement calculation, as kappa takes into account the possibility of the agreement occurring by chance [67]. This criterion was calculated using Eq. 2;

2 $$\mathrm{kappa}=\frac{{p}_{o}-{p}_{e}}{1-{p}_{e}}$$

where ${p}_{o}$ is the relative observed agreement among raters, and ${p}_{e}$ is the hypothetical probability of chance agreement. More detailed information about this equation is revealed by Bonhomme et al. [68]. RMSE is a measure of the differences between the actually observed data and those predicted by the model [65, 69].

3 $$RMSE={\left[\frac{1}{N}\sum_{i=1}^{N}{({Y}_{pred,i}-{Y}_{obs,i})}^{2}\right]}^{0.5}$$

where ${Y}_{obs,i}$ and ${Y}_{obs,i}$ are respectively the *i*th observed and predicted data from N total data. In cases of equal classification accuracies, the models that resulted in higher kappa and lower RMSE values, were selected as the superior models.

### DT-based fuzzy system

In order to define fuzzy membership functions and fuzzy rules implementation, it was necessary to enter the obtained DT structures into the fuzzy model. For designing the antecedent and consequent parts of the fuzzy rules, the branching of DTs was considered. The most suitable trees were selected based on two factors; the highest accuracy, and the simplest structure. Simple structure of DT is very important in defining the rules and membership functions of DT-based fuzzy system. The input variables and their membership functions were defined according to the nodes and their related threshold values on DTs structure.

A Mamdani fuzzy model was designed and implemented based on the structure of the selected DT. The antecedent and consequent parts of the fuzzy rules were developed and adjusted according to the branches and leaves of the DT, respectively. Also, the nodes and threshold values of the DT were set as the input variables and those related membership functions in the fuzzy system. The classification model was designed and implemented in the fuzzy toolbox of MATLAB software.

## Results

### Results of feature selection methods

The large number of input variables encouraged to apply feature selection/reduction methods to extract the most prominent input vector. The CFS method selected 12 features as the input feature vector for prediction of plant type (4 target classes). In the case of crop/weed classification (2 target classes), there were 9 features that selected by CFS algorithm as the input vector. The selected features are presented in Table 1.

**Table 1:** Number of selected features by CFS and PCA feature selection methods

Classification	Selected features
Plant type detection (4 target classes)	std_R, ave_H, ave_S, std_S, ave_As, std_As, ave_Bs, difference_entropy, cluster_prominence_LL, cluster_shade_LL, entropy_HL, sum_entropy_HH
Crop/weed classification (2 target classes)	ave_H, ave_S, ave_As, std_As, ave_Bs, correlation_LL, cluster_shade_LL, homogeneity_HL, sum_entropy_HH

The PCA method produced 18 principal components from the original features for prediction of plant type (4 target classes) as well as for crop/weed classification (2 target classes). Because the PCA method is unsupervised, the principal component don’t vary by the number of target classes.

### Results on DT structure

**Fig. 2:**
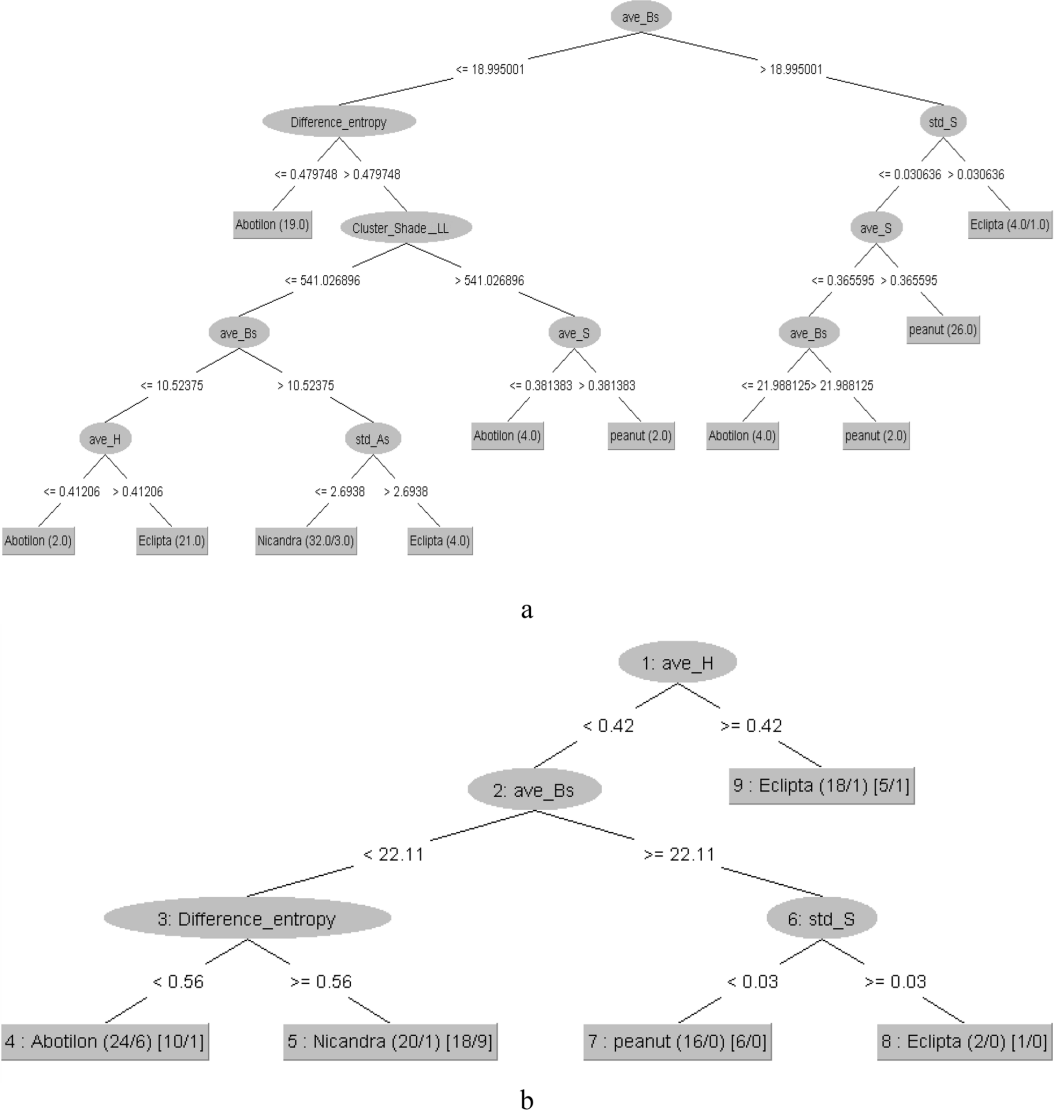
The overall structure of **a** J48-CFS, and **b** REP-CFS, decision trees for classification of plants

Training and testing results of J48, REP and RT classifiers for simultaneously classification of four different plants (4 classes including peanut and three weeds) based on visual characterization are shown in Table 2. The highest classification accuracy (80.83%) for plant type detection in training dataset was obtained when using J48 tree as the classifier in which the CFS selected features were used as inputs. The RMSE and kappa statistics of this tree on training dataset were 0.74 and 0.2855, respectively. The classification accuracy, RMSE and kappa statistics of J48-CFS tree were respectively obtained as 80.56%, 0.2947, and 0.73, when the tree was evaluated on test dataset. The next most successful classifier was REP tree with CFS-selected features. The overall structure of J48-CFS and REP-CFS trees are presented in Fig. 2.

**Table 2:** Performance criteria of DTs for plant type identification

DT	Feature selection/reduction method	Train			Test		
		Kappa	RMSE	Accuracy (%)	Kappa	RMSE	Accuracy (%)
J48	–^a^	0.70	0.3129	77.50	0.64	0.3483	73.61
	CFS	0.74	0.2855	***80.83***	0.73	0.2947	80.56
	PCA	0.39	0.4594	54.17	0.36	0.4859	52.77
REP	–^a^	0.70	0.3135	77.50	0.60	0.3334	75.00
	CFS	0.73	0.3007	80.00	0.72	0.3143	79.17
	PCA	0.38	0.4121	53.33	0.34	0.4930	51.38
RT	–^a^	0.62	0.3764	71.67	0.57	0.3953	68.75
	CFS	0.72	0.3227	79.17	0.70	0.3371	77.27
	PCA	0.40	0.4743	55.00	0.38	0.4556	54.17

Bolditalic value indicates the most accurate DT classifier

^a^No feature selection applied (classification using all input features)

The performance statistics of different DTs for classification of peanut plant from weeds (2 classes) are given in Table 3 for training and testing datasets. As shown, the J48 DT when using CFS method, was the most accurate tree for classification of peanut plant from group of weeds. The classification accuracy, RMSE and kappa values of this tree were 95.56%, 0.1942, and 0.91, respectively. This tree resulted accuracy of 93.75%, a RMSE of 0.1968, and a kappa value of 0.088, when evaluated by the test data. The next four most accurate classifier DTs were J48 without feature selection (J48-all), RT-CFS, REP-CFS, and REP-all trees with classification accuracies of 93.89%, 93.33%, 92.78%, and 92.22%, respectively.

**Table 3:** Performance criteria of DTs for peanut/weed classification

DT	Feature selection/reduction method	Train			Test		
		Kappa	RMSE	Accuracy (%)	Kappa	RMSE	Accuracy (%)
J48	–*	0.88	0.2472	93.89	0.83	0.2791	91.67
	***CFS***	0.91	0.1942	***95.56***	0.88	0.1968	93.75
	PCA	0.63	0.4200	81.67	0.63	0.4210	81.48
REP	–*	0.84	0.2679	92.22	0.82	0.2888	90.91
	CFS	0.86	0.2557	92.78	0.83	0.2895	91.67
	PCA	0.47	0.4653	73.33	0.48	0.4986	74.074
RT	–*	0.70	0.3873	85.00	0.70	0.3849	85.18
	CFS	0.87	0.2582	93.33	0.85	0.2722	92.59
	PCA	0.46	0.5217	72.78	0.42	0.5401	70.83

Bolditalic value indicates the most accurate DT classifier

^*^ No feature selection applied (classification using all input features)

**Fig. 3:**
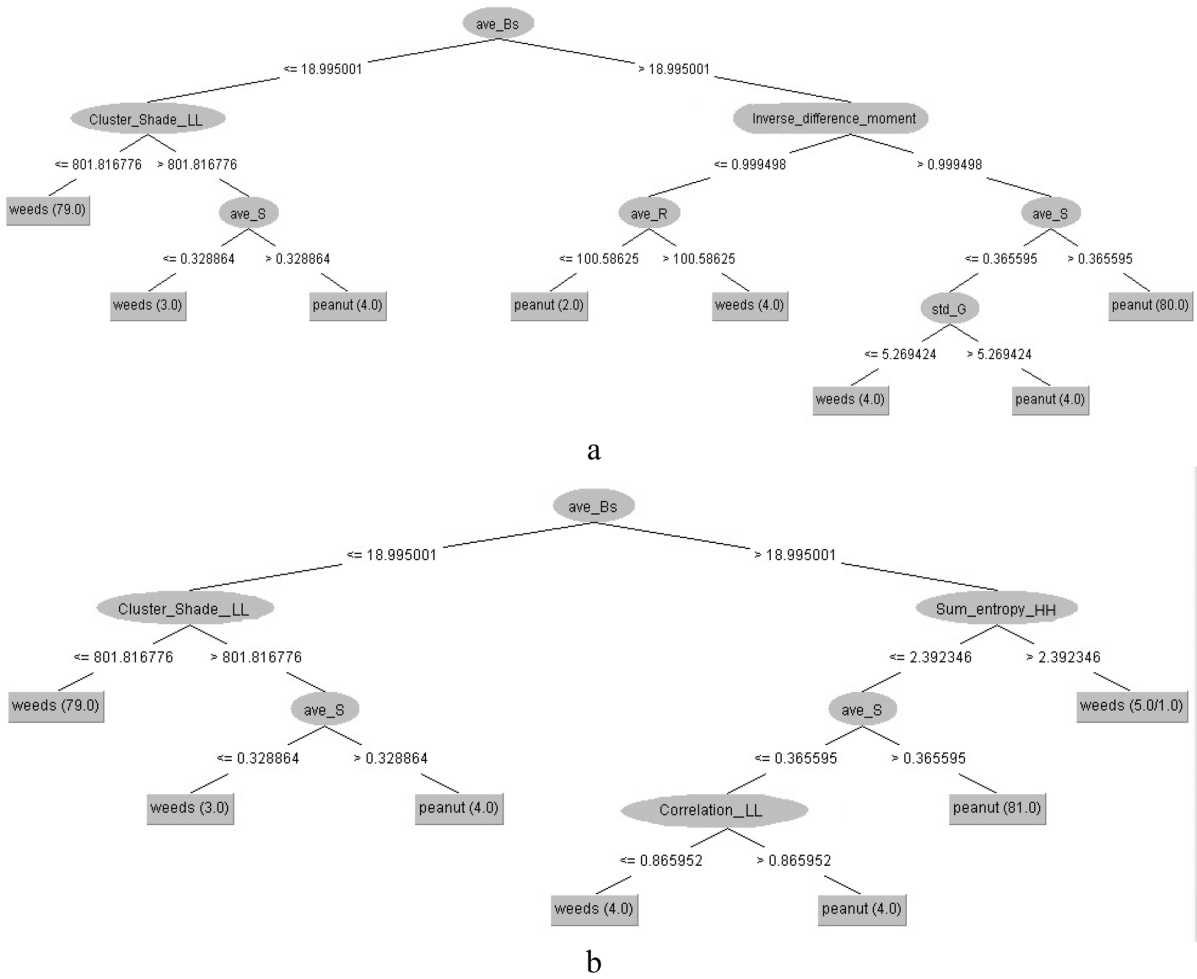
The overall structure of **a** J48-all, and **b** J48-CFS, decision trees for classification of peanut and weeds

**Fig. 4:**
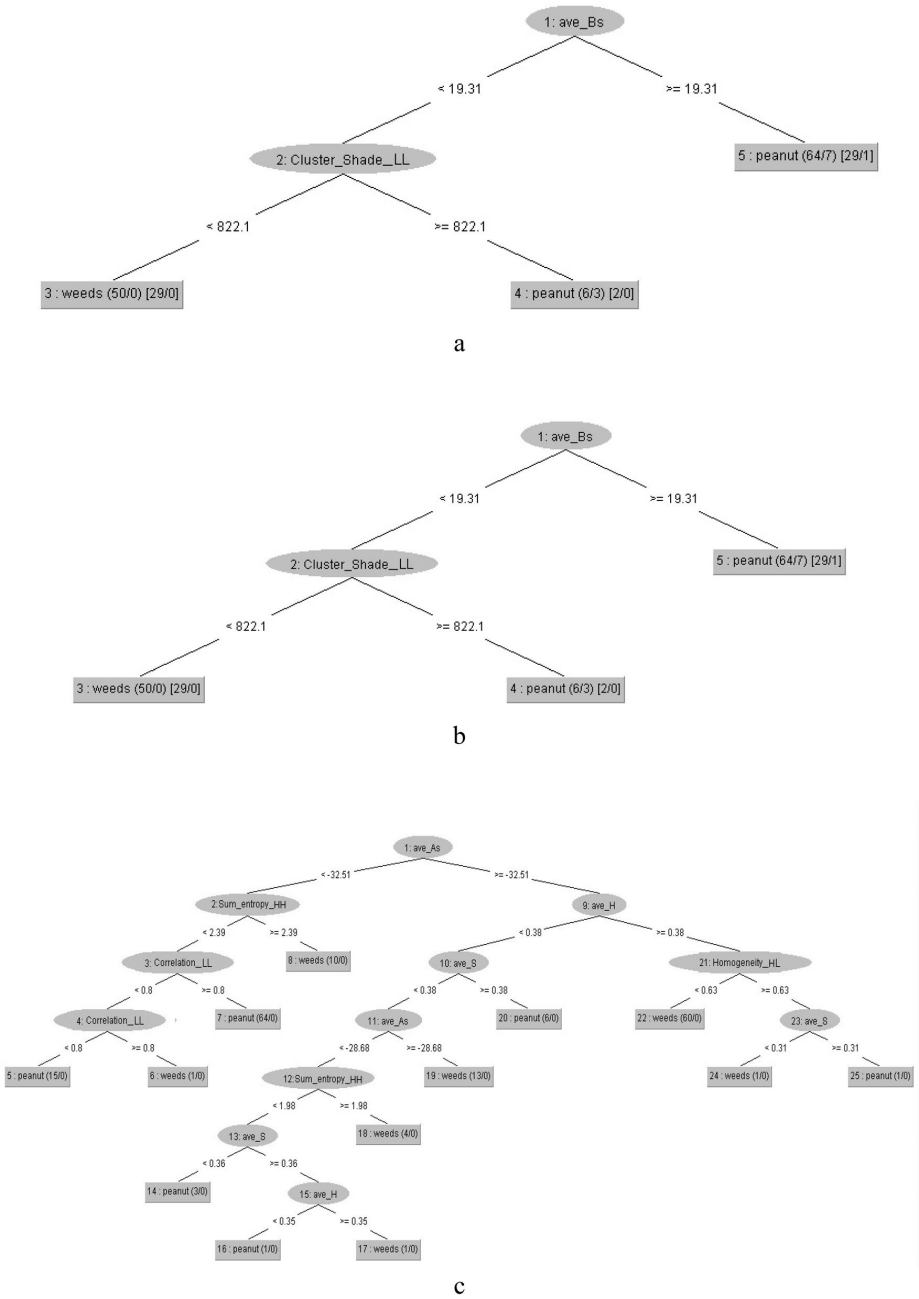
The overall structure of **a** REP-all, **b** REP-CFS, and **c** RT-CFS decision trees for classification of peanut and weeds

Figures 3 and 4 present the overall structures of J48-all, J48-CFS, REP-all, REP-CFS, RT-CFS decision trees for classification of peanut and weeds. As shown, different DTs have resulted in different structures because of their specific algorithm for implementation of trees. It can be observed that, when using either of the CFS and PCA feature selection algorithms, the structures of J48 and REP trees, were simpler compared with RT. The J48-CFS tree obtained with 7 branches, 6 nodes and 7 leaves; whereas J48-PCA tree has 6 branches, 5 nodes and 6 leaves, while the resulted REP-CFS tree had 3 branches, 2 nodes and 3 leaves. Applying RT algorithm resulted in 13 branches with 12 nodes and 13 leaves.

### Results on Fuzzy inference system

**Fig. 5:**
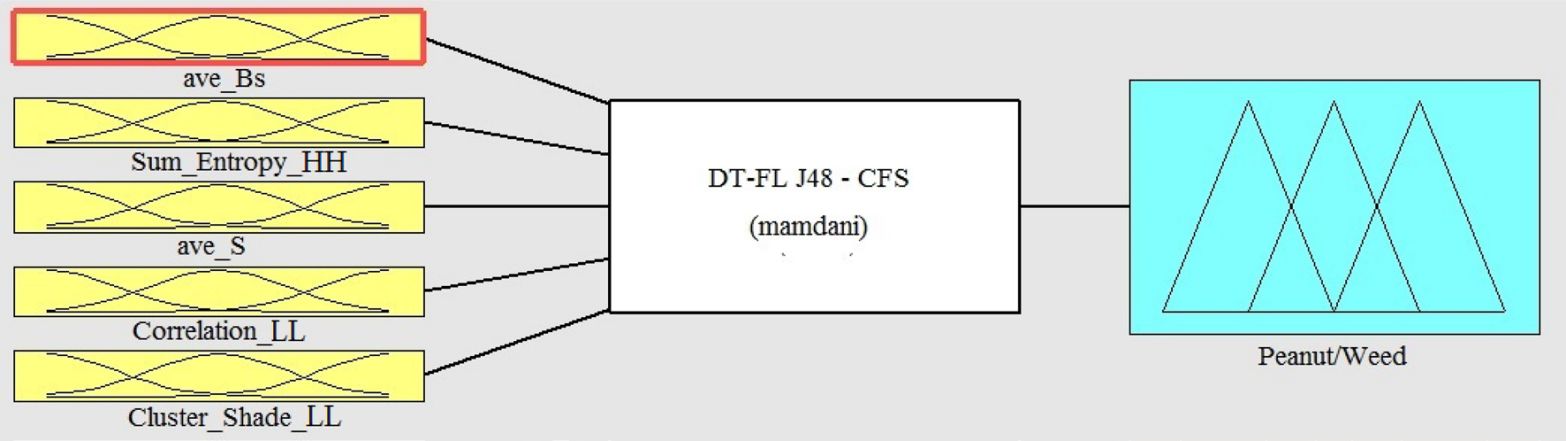
The overall form of J48-CFS based fuzzy model for peanut/weed classification

**Fig. 6:**
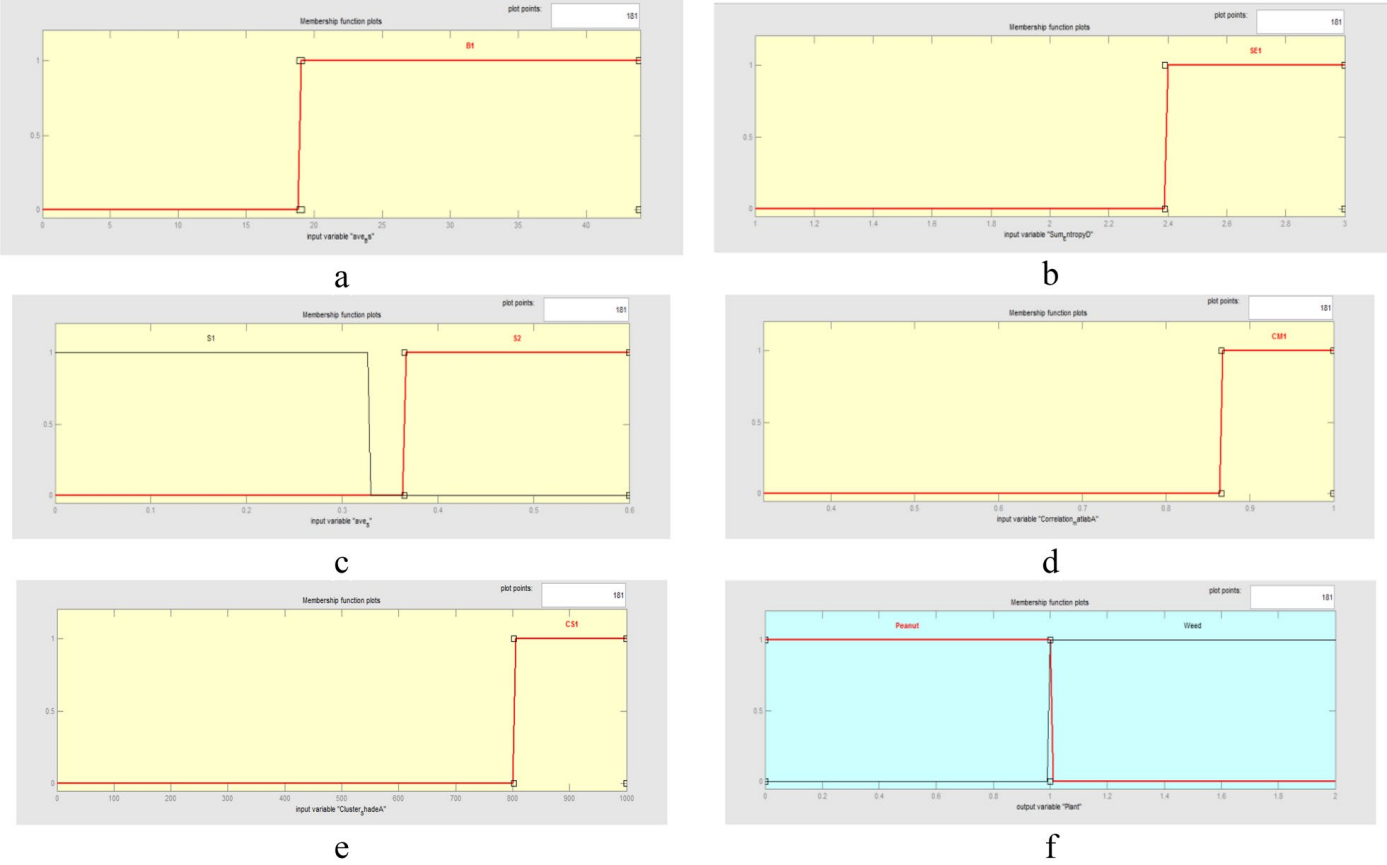
The overall form of fuzzy membership functions for peanut/weed classification using J48-CFS classifier: **a** ave_Bs; **b** sum_entropy_HH; **c** ave_S; **d** correlation_LL; and **e** cluster_shade_LL; **f** Peanut/weed

Figure 5 shows the overall form of fuzzy inference model which was obtained from the J48-CFS decision tree. The membership functions for the fuzzy model variables were defined according to the classifying features and their related threshold values on the branches of the DT. From Fig. 3b, it can be observed that five features including ave_Bs, sum_entropy_HH, ave_S, correlation_LL and cluster_shade_LL were selected as defining factors to setup the structure of J48-CFS tree. Hence, these features were applied to design the antecedent part and to adjust the membership functions of the fuzzy model. Because the simplicity of the trapezoidal membership functions, these functions were used to set all of fuzzy variables. In the case of ave_S, since this feature appeared in two nodes of J48-CFS tree structure, two membership functions were defined accordingly in the fuzzy model. The overall form of the designed membership functions is shown in Fig. 6.

**Fig. 7:**
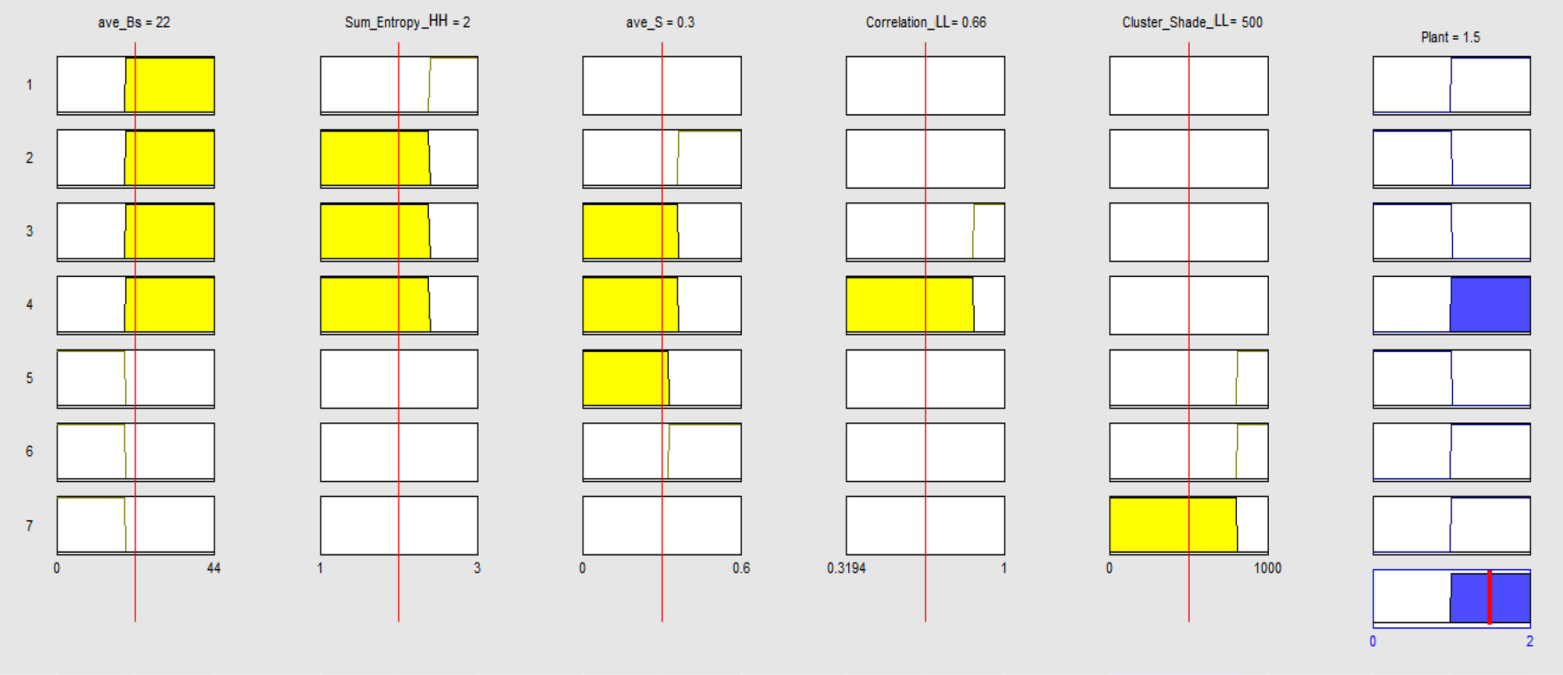
The graphical view of the defined fuzzy rules for peanut/weed classification using J48-CFS classifier

**Fig. 8:**
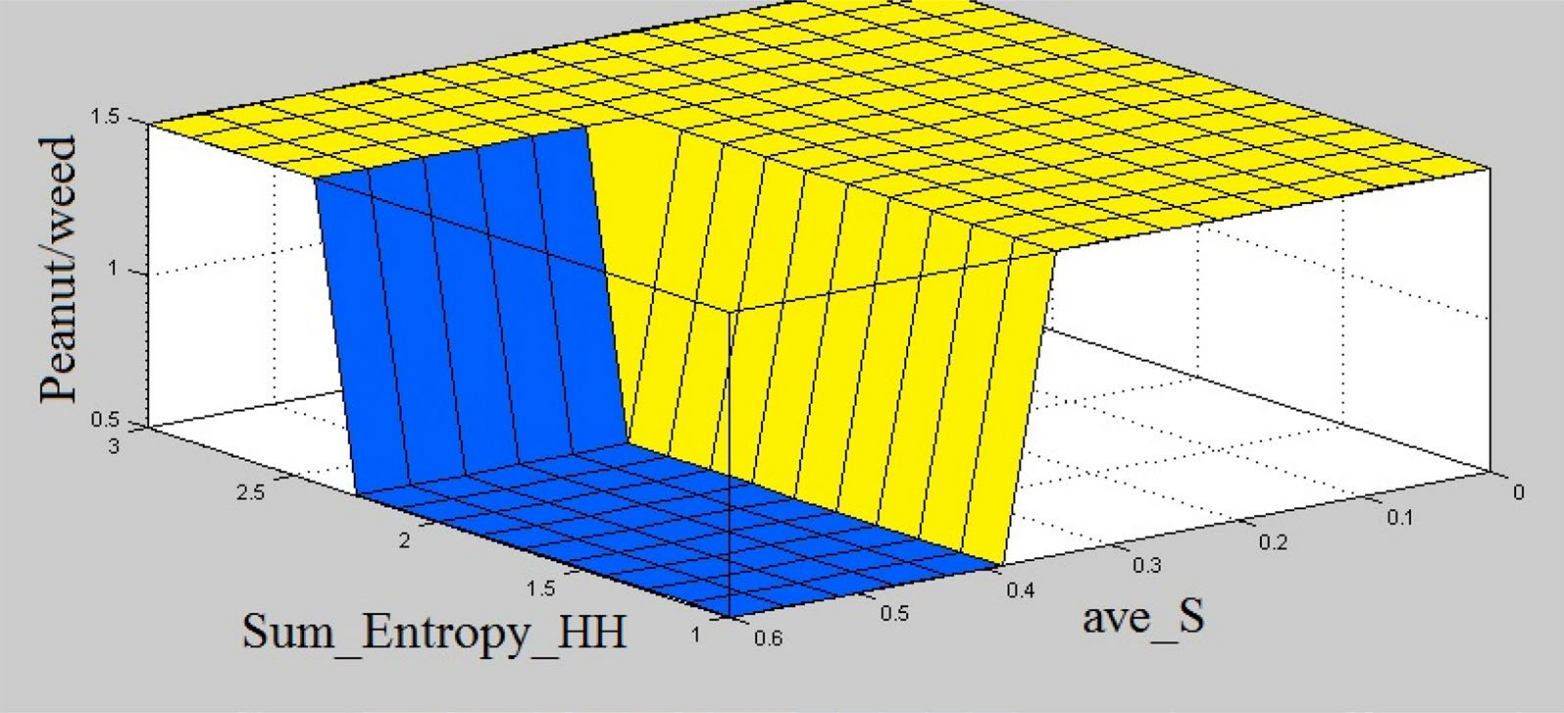
Surface view of fuzzy rules considering different combinations of input variables for peanut/weed classification using J48-CFS classifier

The next step was to implement the rule base of the fuzzy model. The rules were defined in the form of ‘if–then’ statements according to the branches of the J48-CFS tree (Fig. 3b). The defied rules are presented in Table 4. The graphical view of the defined fuzzy rules is shown in Fig. 7. As shown, five features are used in the precedent part of the fuzzy rule base to make a rule. The sixth column in the Figure represents the membership function of the output variable (plant type). The surface view of the fuzzy rules considering different combinations of input variables for peanut/weed classification using J48-CFS classifier is shown in Fig. 8. This figure presents the output value of the model considering ave_S and sum_entropy_HH features as input variables. Plant membership functions, the non-integer values in the model between 0–1 and 1–2 belong to Peanuts and Weeds classes, respectively. It can be observed that if the value of ave_S is greater than 0.36 (which is the threshold value of the mentioned variable on its related fuzzy membership function), the quality value would be 0 which represents Peanuts class. In the case of sum_entropy_HH feature, if the value is less than 2.39, then the output value depends on the threshold value of correlation_LL feature. For sum_entropy_HH values higher than 2.36, the output value will be higher than 1 which corresponds to Weeds class.

**Table 4:** Fuzzy rules for peanut/weed classification using J48-CFS classifier

1. If (ave_Bs is B1) and (sum_entropy_HH is SE1) then (Plant is Weed)
2. If (ave_Bs is B1) and (sum_entropy_HH is not SE1) and (ave_S is S2) then (Plant is Peanut)
3. If (ave_Bs is B1) and (sum_entropy_HH is not SE1) and (ave_S is not S2) and (correlation_LL is CM1) then (Plant is Peanut)
4. If (ave_Bs is B1) and (sum_entropy_HH is not SE1) and (ave_S is not S2) and (correlation_LL is not CM1) then (Plant is Weed)
5. If (ave_Bs is not B1) and (ave_S is S1) and (cluster_shade_LL is CS1) then (Plant is Peanut)
6. If (ave_Bs is not B1) and (ave_S is not S1) and (cluster_shade_LL is CS1) then (Plant is Weed)
7. If (ave_Bs is not B1) and (cluster_shade_LL is not CS1) then (Plant is Weed)

**Fig. 9:**
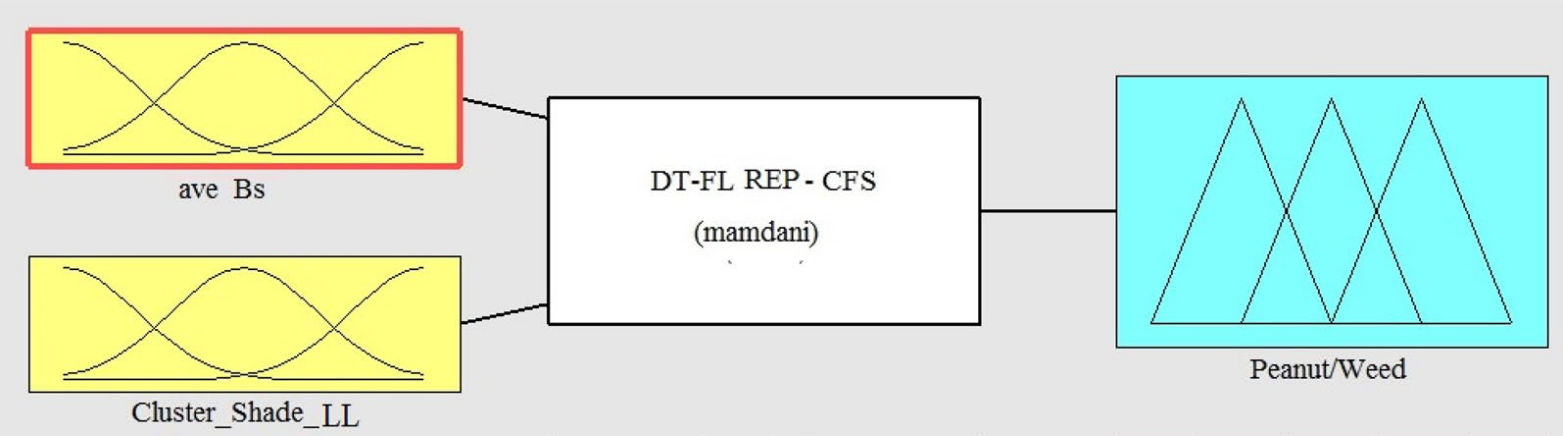
The overall form of REP-CFS based fuzzy model for peanut/weed classification

**Fig. 10:**
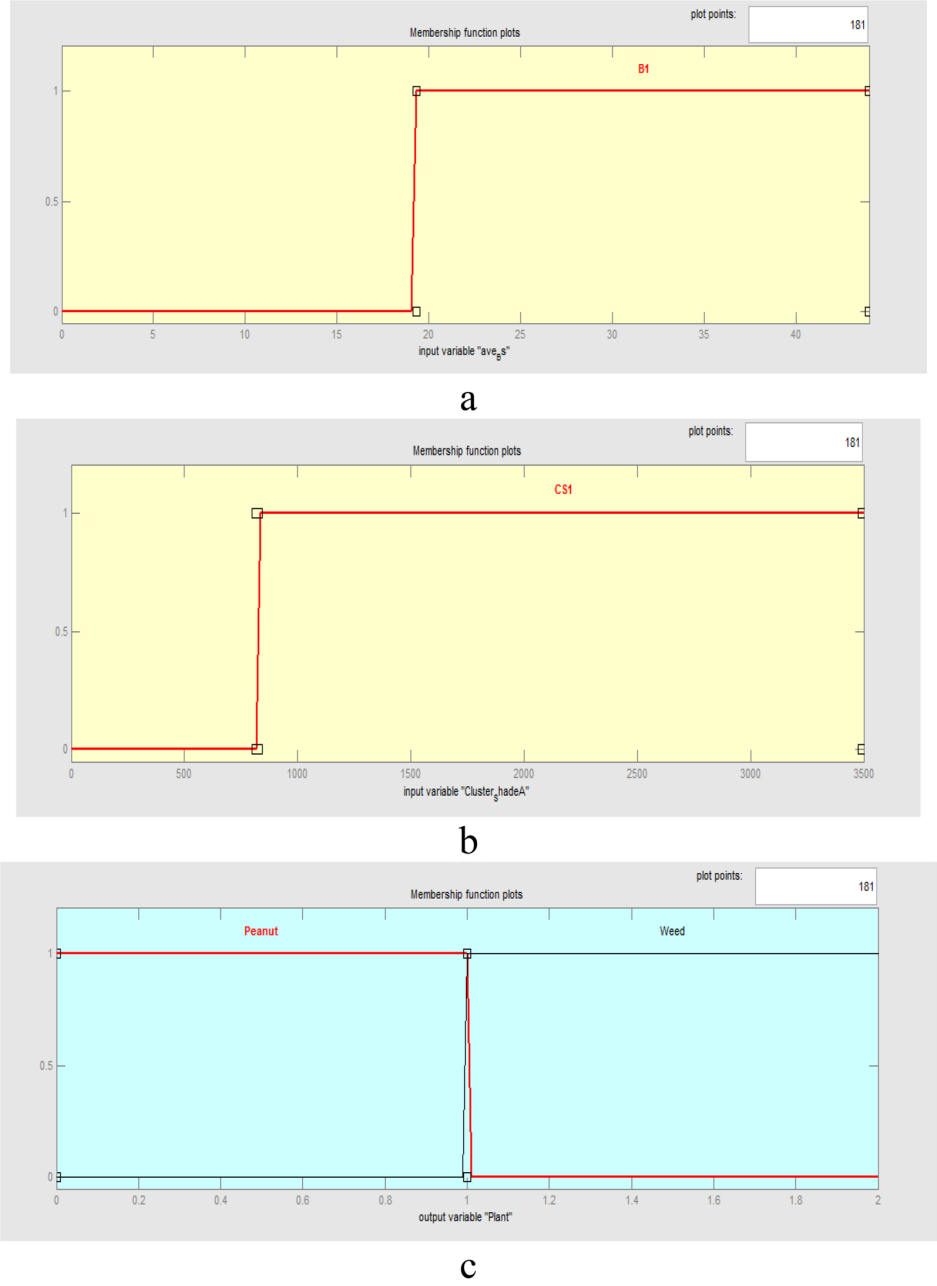
The overall form of fuzzy membership functions for peanut/weed classification using REP-CFS classifier: **a** ave_Bs; **b** cluster_shade_LL; **c** Peanut/weed

The overall form of the fuzzy inference system for peanuts and weeds using REP-CFS classification method is shown in Fig. 9. As can be seen, REP-CFS algorithm gave a simpler fuzzy model as compared with J48-CFS classifier. Here, the fuzzy inference system has only two input variables. The membership functions for the variables in the fuzzy model were obtained according to the defining features and their related threshold values on the branches of the REP-CFS classifier. The range of each membership function was set based on the maximum and minimum values of the related features in the classifying DT. Considering Fig. 4b, two features namely ave_Bs (average of a* chromatic component in L*a*b space) and cluster_shade_LL (the cluster shade feature extracted from approximation coefficient of the one-level wavelet decomposed image) were used in the structure of REP-CFS classifier. The combinations of these features and their related threshold values on the branches of REP-CFS classifying tree were used to set the antecedent part of the fuzzy model. The overall form of the designed fuzzy membership functions are presented in Fig. 10.

The rule base of the fuzzy model using REP-CFS classification method is shown in Table 5. According to Fig. 4b, three interconnections were created on the structure of REP-CFS tree to implement the fuzzy rule base.

**Table 5:** Fuzzy rules defined for peanut/weed classification using REP-CFS classifier

1. If (ave_Bs is B1) then (Plant is Peanut)
2. If (ave_Bs is not B1) and (cluster_shade_LL is CS1) then (Plant is Peanut)
3. If (ave_Bs is not B1) and (cluster_shade_LL is not CS1) then (Plant is Weed)

**Fig. 11:**
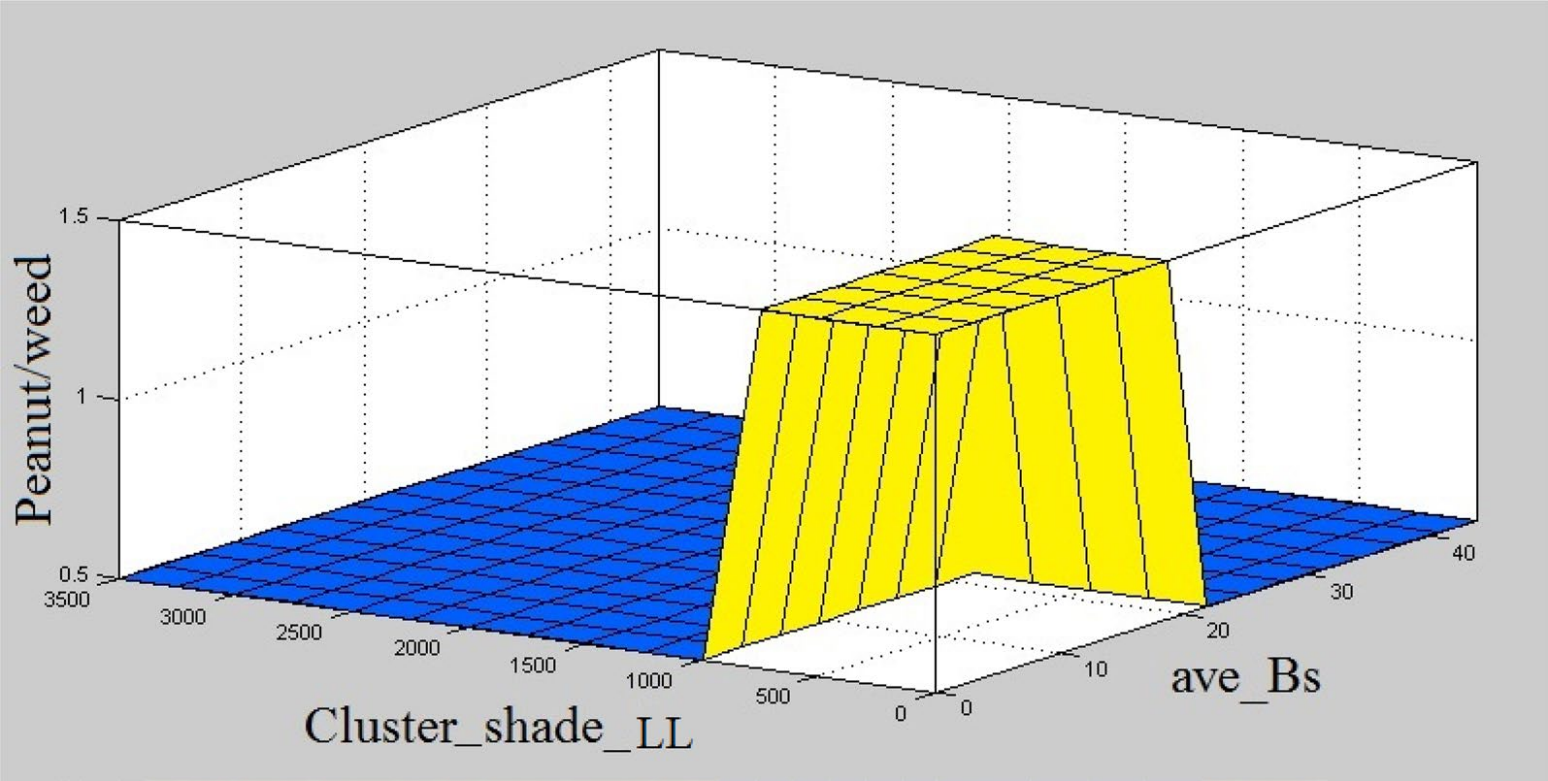
Surface view of fuzzy rules considering different combinations of input variables for classification of peanuts and weeds using REP-CFS classifier

The surface view of the defined fuzzy rules for peanuts and weeds classification using REP-CFS classifier is shown in Fig. 11. This figure gives a 3-D view of the fuzzy model and shows how different combinations of the input variables result in an output class. In Fig. 11, it can be seen that when the values of ave_Bs and cluster_shade_LL features are respectively in the range of 0–19.31 and 0–822.1, the output value of the model is greater than 1 which shows the plant is weed. As correlation_LL value exceeds 822.1, the product grade value lies between 0 and 1 that is the range corresponding to Peanuts. The same result is obtained when the value of ave_Bs feature surpasses 19.31.

## Discussions

Considering the results obtained for plant type identification by DT (Table 2), DTs have achieved low classification accuracies for plant type identification making them unsuitable to be applied for development of a fuzzy classifier. On the other side, since different species of weeds are there in the peanut fields, it is required for a weeding robot to classify main crop from weeds. Therefore, the capability of DT was investigated for crop/weed classification purpose. In this case, DTs with CFS feature selection method resulted in the highest classification accuracies making them the most suitable trees for fuzzy classifier development.

Consequently, it can be concluded from Fig. 2b that the REP-CFS tree provides a more simple structure for data classification compared with RT-CFS and J48-CFS tree. Whereas, J48 tree when combined with CFS feature selection method (J48-CFS) gave the highest accuracy. Although simplicity is not a criterion to be considered for comparing the performance of DT classifier, but, from the point of view of fuzzy model development based on DT structure, the simplicity of DT structure is a promising option that can help to achieve a simpler fuzzy model. Regarding that development of automated plant weeding system is one of the most important practical fields of fuzzy models, the use of a less complex DT-based fuzzy system, provided that the desired accuracy is guaranteed, can result in a higher performance for automatic weeding systems. Therefore, considering two important criteria of DT simplicity and overall classification accuracy, two DT structures of J48-CFS (because of having the highest accuracy) and REP-CFS (because of having the simplest structure and satisfactory accuracy) were selected for developing fuzzy models.

The J48-CFS tree has a very complex structure (Fig. 2a) which makes it inappropriate for developing a fuzzy system for plant type identification. The REP-CFS has very simpler structure (Fig. 2b) than J48 tree. The REP-CFS structure has 5 branches, 4 nodes and 5 leaves; while the J48-CFS tree obtained with 11 branches, 11 nodes and 10 leaves.

For all of the three evaluated DTs, applying PCA feature selection method has resulted in lowest classification accuracy. Therefore, DTs with PCA feature selection method were not used in further steps of fuzzy model implementation.

In the structure of J48-CFS decision tree, five features including ave_Bs (average of b* chromatic component in L*a*b space), sum_entropy_HH (Summation of entropy values extracted from diagonal subband of the one-level wavelet decomposed image), ave_S (average of Saturation component in HSV space), correlation_LL (the correlation feature extracted from approximation subband of the one-level wavelet decomposed image) and cluster_shade_LL (the cluster shade feature extracted from approximation subband of the one-level wavelet decomposed image) were appeared in the nodes positions. Peanuts and weeds were classified according to combination of these features, the related threshold values, appearance order and position on DTs structure.

Among the contributing features for forming the structure of the J48-CFS tree, two features (ave_Bs and ave_S) were obtained from the color information of the images and three features (sum_entropy_HH, correlation_LL and cluster_shade_LL) from the wavelet analysis of the images. This proves that the color and wavelet textural information of the samples are both significant and effective in the distinguishment of peanuts from weeds. The ave_S feature was appeared two times in the structure of J48-CFS tree which may implies the significance of this feature for the product classification. According to the branching of the J48-CFS tree, it is evident that some features were not included in forming a numbers of fuzzy rules.

Considering the shape of the plant membership functions (Fig. 7), the decimal values lower than 1 and 2 in the functions belong to Peanut and Weed classes, respectively. The non-integrity of the output values is due to the uncertain nature of the fuzzy-logic-based models. In these situations, if the fuzzy model is used in the form of an online classifying system, the value of the output variable will be rounded to the nearest integer number.

In the case of REP-CFS tree, only two features namely ave_Bs (average of b* chromatic component in L*a*b space) and cluster_shade_LL (the cluster shade feature extracted from approximation subband of the one-level wavelet decomposed image) were used for construction of the tree (Fig. 4a). Considering its simplicity advantage along with high accuracy and low RMSE values, the capability of REP-CFS tree for classification becomes more prominent. This leads to the production of a precise fuzzy model with very simple structure. Due to the simplicity of utilized membership functions in REP-CFS based fuzzy classifier, the developed fuzzy model would have a high calculation and execution speed.

The low number of rules accelerates the execution of the fuzzy model when classifying peanuts and weeds. From Table 5, it can be observed that the antecedent part of the rule base is not complex compared with that of J48 trees. The antecedent parts were adjusted by the combination of up to two input variables. Due to the simplicity of its utilized membership functions, the developed fuzzy model would have a high calculation and execution speed along with high accuracy. The high reliability, speed of response, and accuracy of such model are important especially when the model is applied in the form of real-time classifying systems.

A very important advantage of DT based fuzzy classifier over classical and ensemble models appears when the classifier is used as the controller of an automatic weed removal system. Binary controllers get in trouble in the boundary regions of objects especially where the main plant and weed are highly overlapped. Hard splits between different regions, showing different plants, can induce several on–off shocks to the operators. Fuzzy controllers can overcome this problem by converting hard splits to soft splits because of the angular shape of their rule surfaces in overlapping regions.

In addition, such a robotic weeding system should be able to operate in a working condition which cannot be completely controlled like as laboratory conditions. In such cases, DT-fuzzy based control systems are superior to binary models, because the DT-fuzzy systems are less sensitive to probable environmental and working noises.

The obtained results in this study confirm the results of previous studies reporting the successful application of fuzzy logic systems for weed-crop classification. Herrera et al. [37], reported an accuracy of 92.9% for classification of monocots and dicots weeds at early stage of growth based on shape descriptors using fuzzy multicriteria decision making (FMDM). A similar accuracy was reported for real-time weed detecting in sugarcane field based on leaf textures and fuzzy technique [38]. Although the application of DT-fuzzy system has not been reported for weed detection, however DT-fuzzy models were successfully applied for some other classification purposes. The classification accuracy of a combined logic ‐fuzzy logic model was reported 91.74% for classification of the dried figs where the structure of REP tree was used for fuzzy system development [42]. A J48 DT-fuzzy system was also applied by Goel, Sehgal [34] for ripeness estimation of tomatoes with a classification accuracy of 94.29%.

## Conclusions

A fuzzy computer vision system was developed in this study to classify peanut plant from weeds based on image color and wavelet features using a combination of DT and fuzzy logic. PCA and CFS strategies to reduce the number of DT input features and to select the most significant ones. J48 and REP trees developed using the CFS selected date, were the most accurate DTs for crop-weed differentiation. The crop-weed classification accuracy of J48-CFS and REP-CFS models was achieved 95.56% and 92.78%. These two trees were also have simple configurations made them appropriate to design fuzzy sets based on their schemes. The “if–then” statements of fuzzy rules were established according to nodes, branches, leaves and thresholds which provided in DTs. It can be claimed from the results of this study, that the fuzzy DT classification technique can be applied successfully in a computer vision system for intelligent classification of main crop from weeds in peanut fields.

## Data Availability

The primary images that were acquired from peanut fields and the extracted features datasets used and/or analyzed during the current study are available from the corresponding author on reasonable request. All the other data generated or analyzed during this study are included within this article.

## References

[1] Hussain M, Farooq S, Merfield C, Jabran K (2018). Mechanical weed control. Non-Chemical Weed Control.

[2] Parameswari Y, Srinivas A (2017). Weed management in rice—a review. Int J Appl Pure Sci Agric..

[3] Ahmad B, Taufik M. Development of an automated mechanical intra-row weeder for vegetable crops. 2012.

[4] Pérez-Ruiz M, Slaughter D, Gliever C, Upadhyaya S (2012). Automatic GPS-based intra-row weed knife control system for transplanted row crops. Comput Electron Agric.

[5] Sabzi S, Abbaspour-Gilandeh Y, Javadikia H (2017). The use of soft computing to classification of some weeds based on video processing. Appl Soft Comp.

[6] Pantazi X-E, Moshou D, Bravo C (2016). Active learning system for weed species recognition based on hyperspectral sensing. Biosys Eng.

[7] Christensen S, Søgaard HT, Kudsk P, Nørremark M, Lund I, Nadimi ES (2009). Site-specific weed control technologies. Weed Res.

[8] Dammer KH (2016). Real-time variable-rate herbicide application for weed control in carrots. Weed Res.

[9] Barbedo JGA, Koenigkan LV, Santos TT (2016). Identifying multiple plant diseases using digital image processing. Biosys Eng.

[10] Cubero S, Albert F, Prats-Moltalbán JM, Fernández-Pacheco DG, Blasco J, Aleixos N (2018). Application for the estimation of the standard citrus colour index (CCI) using image processing in mobile devices. Biosys Eng.

[11] Dutta MK, Issac A, Minhas N, Sarkar B (2016). Image processing based method to assess fish quality and freshness. J Food Eng.

[12] Hosseininia SAR, Kamani MH, Rani S (2017). Quantitative determination of sunset yellow concentration in soft drinks via digital image processing. J Food Meas Charact.

[13] Agin O, Taner A (2015). Determination of weed intensity in wheat production using image processing techniques. Anadolu Tarim Bilimleri Dergisi.

[14] Bakhshipour A, Jafari A (2018). Evaluation of support vector machine and artificial neural networks in weed detection using shape features. Comput Electron Agric.

[15] Tang J-L, Chen X-Q, Miao R-H, Wang D (2016). Weed detection using image processing under different illumination for site-specific areas spraying. Comput Electron Agric.

[16] Golzarian MR, Frick RA (2011). Classification of images of wheat, ryegrass and brome grass species at early growth stages using principal component analysis. Plant Methods.

[17] Gao J, Nuyttens D, Lootens P, He Y, Pieters JG (2018). Recognising weeds in a maize crop using a random forest machine-learning algorithm and near-infrared snapshot mosaic hyperspectral imagery. Biosys Eng.

[18] Ghasemloo N, Mobasheri M, Rezaei Y (2011). Vegetation species determination using Spectral Characteristics and Artificial Neural Network (SCANN). J Agric Sci Technol.

[19] Righetto AJ, Ramires TG, Nakamura LR, Castanho PL, Faes C, Savian TV (2018). Predicting weed invasion in a sugarcane cultivar using multispectral image. J Appl Stat..

[20] Lameski P, Zdravevski E, Trajkovik V, Kulakov A. Weed detection dataset with RGB images taken under variable light conditions. In: International Conference on ICT Innovations: Springer; 2017. p. 112–9.

[21] Doering D, Vizzotto M, Bredemeier C, da Costa C, Henriques R, Pignaton E (2016). MDE-based development of a multispectral camera for precision agriculture. IFAC-PapersOnLine.

[22] Yamuna S, Devi LP, Yamunai S. Human face recognition under varying illumination condition using wavelet transform. In: Intelligent Computing Applications (ICICA), 2014 International Conference on: IEEE; 2014. p. 280–4.

[23] Wazarkar S, Keshavamurthy BN (2018). A survey on image data analysis through clustering techniques for real world applications. J Vis Commun Image Represent.

[24] Omrani E, Khoshnevisan B, Shamshirband S, Saboohi H, Anuar NB, Nasir MHNM (2014). Potential of radial basis function-based support vector regression for apple disease detection. Measurement.

[25] Vyas A, Yu S, Paik J (2018). Multiscale transforms with application to image processing.

[26] Kolekar MKH, Raja GL, Sengupta S. An introduction to wavelet-based image processing and its applications. In: Computer Vision: Concepts, Methodologies, Tools, and Applications. IGI Global; 2018. p. 110–28.

[27] Khoje S (2018). Appearance and characterization of fruit image textures for quality sorting using wavelet transform and genetic algorithms. J Texture Stud.

[28] Wang S, Yang X, Zhang Y, Phillips P, Yang J, Yuan T-F (2015). Identification of green, oolong and black teas in China via wavelet packet entropy and fuzzy support vector machine. Entropy.

[29] Lopez MEA (2018). A monitoring and threat detection system using stream processing as a virtual function for big data.

[30] Veloso A, Meira Jr W, Zaki MJ. Lazy associative classification. Data Mining, 2006 ICDM'06 Sixth International Conference on: IEEE; 2006. p. 645–54.

[31] Geurts P, Ernst D, Wehenkel L (2006). Extremely randomized trees. Machine learning.

[32] Zadeh LA (1965). Information and control. Fuzzy Sets.

[33] Kamila NK, Mallick PK. A novel fuzzy logic classifier for classification and quality measurement of apple fruit. In: Handbook of research on emerging perspectives in intelligent pattern recognition, analysis, and image processing. IGI Global; 2016. p. 367–82.

[34] Goel N, Sehgal P (2015). Fuzzy classification of pre-harvest tomatoes for ripeness estimation–An approach based on automatic rule learning using decision tree. Appl Soft Comp.

[35] Guetova M, Hölldobler S, Störr H-P. Incremental fuzzy decision trees. In: Annual Conference on Artificial Intelligence. Berlin: Springer; 2002. p. 67–81.

[36] Yang C-C, Prasher SO, Landry J-A, Ramaswamy HS (2003). Development of an image processing system and a fuzzy algorithm for site-specific herbicide applications. Precis Agric.

[37] Herrera PJ, Dorado J, Ribeiro Á (2014). A novel approach for weed type classification based on shape descriptors and a fuzzy decision-making method. Sensors.

[38] Sujaritha M, Annadurai S, Satheeshkumar J, Sharan SK, Mahesh L (2017). Weed detecting robot in sugarcane fields using fuzzy real time classifier. Comput Electron Agric.

[39] Dabiri A, Nazari M, Butcher EA. Adaptive neural-fuzzy inference system to control dynamical systems with fractional order dampers. In: American Control Conference (ACC), 2017. IEEE; 2017. p. 1972–7.

[40] Kangrang A, Jiwlong W (2016). Fuzzy-GA Approach for Estimating Rainfall over Upper Chi-Mun Basins of Thailand. J Agric Sci Technol.

[41] Ayed AB, Benhammouda M, Halima MB, Alimi AM. Random forest ensemble classification based fuzzy logic. In: Ninth International Conference on Machine Vision (ICMV 2016): International Society for Optics and Photonics; 2017. p. 103412B.

[42] Banakar A, Zareiforoush H, Baigvand M, Montazeri M, Khodaei J, Behroozi-Khazaei N (2017). Combined application of decision tree and fuzzy logic techniques for intelligent grading of dried figs. J Food Process Eng.

[43] Omid M (2011). Design of an expert system for sorting pistachio nuts through decision tree and fuzzy logic classifier. Exp Sys Appl.

[44] Bakhshipour A, Jafari A, Nassiri SM, Zare D (2017). Weed segmentation using texture features extracted from wavelet sub-images. Biosys Eng.

[45] Forero Vargas MG, Herrera-Rivera S, Ávila-Navarro J, Franco CA, Rasmussen J, Nielsen J. Color Classification Methods for Perennial Weed Detection in Cereal Crops. In: Iberoamerican Congress on Pattern Recognition 2019.

[46] Tiwari O, Goyal V, Kumar P, Vij S. An experimental set up for utilizing convolutional neural network in automated weed detection. In: 2019 4th International Conference on Internet of Things: Smart Innovation and Usages (IoT-SIU): IEEE; 2019. p. 1–6.

[47] Meyer GE, Neto JC (2008). Verification of color vegetation indices for automated crop imaging applications. Comput Electron Agric.

[48] Kim T-H, Youn J-I (2013). Development of a Smartphone-based Pupillometer. J Opt Soc Korea.

[49] Pu Y-Y, Zhao M, O’Donnell C, Sun D-W (2018). Nondestructive quality evaluation of banana slices during microwave vacuum drying using spectral and imaging techniques. Drying Technol.

[50] Castro W, Oblitas J, De-La-Torre M, Cotrina C, Bazán K, Avila-George H (2019). Classification of Cape gooseberry fruit according to its level of ripeness using machine learning techniques and different color spaces. IEEE Access.

[51] Haralick RM (1979). Statistical and structural approaches to texture. Proc IEEE.

[52] Hall-Beyer M. GLCM texture: a tutorial. National Council on Geographic Information and Analysis Remote Sensing Core Curriculum. 2000.

[53] Park B, Chen Y (2001). Co-occurrence matrix texture features of multi-spectral images on poultry carcasses. J Agric Eng Res.

[54] Kurtulmuş F, Ünal H (2015). Discriminating rapeseed varieties using computer vision and machine learning. Exp Syst Appl.

[55] Rahimi-Ajdadi F, Gilandeh YA, Mollazade K, Hasanzadeh RP (2016). Application of machine vision for classification of soil aggregate size. Soil Till Res.

[56] Gupta D, Choubey S (2015). Discrete wavelet transform for image processing. Int J Emerg Tech Adv Eng.

[57] Saeys Y, Inza I, Larrañaga P (2007). A review of feature selection techniques in bioinformatics. Bioinform.

[58] Khalid S, Khalil T, Nasreen S. A survey of feature selection and feature extraction techniques in machine learning. In: 2014 Science and Information Conference: IEEE; 2014. p. 372–8.

[59] Farahnakian F, Mozayani N. Evaluating feature selection techniques in simulated soccer multi agents system. In: 2009 International Conference on Advanced Computer Control: IEEE; 2009. p. 107–10.

[60] Caballero D, Caro A, Rodríguez PG, Durán ML, del Mar ÁM, Palacios R (2016). Modeling salt diffusion in Iberian ham by applying MRI and data mining. J Food Eng.

[61] Lu W, Li Z, Chu J (2017). A novel computer-aided diagnosis system for breast MRI based on feature selection and ensemble learning. Comput Biol Med.

[62] Wu X, Kumar V, Quinlan JR, Ghosh J, Yang Q, Motoda H (2008). Top 10 algorithms in data mining. Knowl Inf Syst.

[63] Dhakate PP, Patil S, Rajeswari K, Abin D (2014). Preprocessing and Classification in WEKA using different classifiers. Inter J Eng Res Appl.

[64] Imah E, Rahayu Y, Wintarti A. Plant leaf recognition using competitive based learning algorithm. In: IOP Conference Series: Materials Science and Engineering: IOP Publishing; 2018. p. 012058.

[65] Zareiforoush H, Minaei S, Alizadeh MR, Banakar A (2016). Qualitative classification of milled rice grains using computer vision and metaheuristic techniques. J Food Sci Tech.

[66] Arakeri MP, Kumar BV, Barsaiya S, Sairam H. Computer vision based robotic weed control system for precision agriculture. In: 2017 International Conference on Advances in Computing, Communications and Informatics (ICACCI): IEEE; 2017. p. 1201–5.

[67] Dharani M, Sreenivasulu G (2018). Land classification of land sat multispectral image using principal component analysis and morphological operations. J Adv Res Dyn Control Syst.

[68] Bonhomme V, Castets M, Morel J, Gaucherel C (2015). Introducing the vectorial Kappa: An index to quantify congruence between vectorial mosaics. Ecol Indicators.

[69] Gupta S, Verma N (2016). Comparative analysis of classification algorithms using WEKA tool. Int J Sci Eng Res..

